# Supplementary material for: Modeling glioblastoma heterogeneity as a dynamic network of cell states
Source: Mol Syst Biol. 2021 Sep 16;17(9):e10105. doi: 10.15252/msb.202010105 (PMC8444284; doi:10.15252/msb.202010105)
Supplement: Supplementary file 6 — Source Data for Figure 5 [file MSB-17-e10105-s004.zip › Figure5A_sourcedata/GSEA_3017/nosigdb_stateF.GseaPreranked.1622795015644/RICHARDS_NOWAKOWSKI_SCIENCE_2017_OPC_UPREG.html]

Details for gene set RICHARDS\_NOWAKOWSKI\_SCIENCE\_2017\_OPC\_UPREG[GSEA]

|  || Dataset | state33017 |
| Phenotype | NoPhenotypeAvailable |
| Upregulated in class | na\_pos |
| GeneSet | RICHARDS\_NOWAKOWSKI\_SCIENCE\_2017\_OPC\_UPREG |
| Enrichment Score (ES) | 0.42289326 |
| Normalized Enrichment Score (NES) | 2.8763812 |
| Nominal p-value | 0.0 |
| FDR q-value | 0.0 |
| FWER p-Value | 0.0 |
Table: GSEA Results Summary

  

Fig 1: Enrichment plot: RICHARDS\_NOWAKOWSKI\_SCIENCE\_2017\_OPC\_UPREG      
 Profile of the Running ES Score & Positions of GeneSet Members on the Rank Ordered List

  

| PROBE | GENE SYMBOL | GENE\_TITLE | RANK IN GENE LIST | RANK METRIC SCORE | RUNNING ES | CORE ENRICHMENT || 1 | MT3 |  |  | 1 | 2.229 | 0.0303 | Yes |
| 2 | ZMAT3 |  |  | 10 | 1.568 | 0.0481 | Yes |
| 3 | S100B |  |  | 20 | 1.167 | 0.0600 | Yes |
| 4 | CSPG5 |  |  | 30 | 1.044 | 0.0701 | Yes |
| 5 | RPS27L |  |  | 33 | 1.025 | 0.0833 | Yes |
| 6 | ITPR2 |  |  | 34 | 1.024 | 0.0974 | Yes |
| 7 | TNFRSF21 |  |  | 36 | 1.016 | 0.1110 | Yes |
| 8 | EDNRB |  |  | 44 | 0.957 | 0.1209 | Yes |
| 9 | KCNQ1OT1 |  |  | 65 | 0.855 | 0.1232 | Yes |
| 10 | GPR155 |  |  | 70 | 0.832 | 0.1328 | Yes |
| 11 | UBL3 |  |  | 88 | 0.778 | 0.1354 | Yes |
| 12 | LRP1 |  |  | 92 | 0.765 | 0.1445 | Yes |
| 13 | PON2 |  |  | 93 | 0.764 | 0.1551 | Yes |
| 14 | LHFPL3 |  |  | 98 | 0.755 | 0.1636 | Yes |
| 15 | GRIA4 |  |  | 99 | 0.754 | 0.1740 | Yes |
| 16 | GRIA2 |  |  | 105 | 0.743 | 0.1819 | Yes |
| 17 | LUZP2 |  |  | 106 | 0.742 | 0.1921 | Yes |
| 18 | B2M |  |  | 110 | 0.736 | 0.2009 | Yes |
| 19 | SCG3 |  |  | 130 | 0.698 | 0.2015 | Yes |
| 20 | CADM2 |  |  | 137 | 0.687 | 0.2081 | Yes |
| 21 | FIBIN |  |  | 147 | 0.673 | 0.2131 | Yes |
| 22 | TSPAN7 |  |  | 148 | 0.671 | 0.2224 | Yes |
| 23 | FAM84A |  |  | 157 | 0.650 | 0.2275 | Yes |
| 24 | ITM2C |  |  | 160 | 0.649 | 0.2355 | Yes |
| 25 | CSGALNACT1 |  |  | 172 | 0.628 | 0.2390 | Yes |
| 26 | ST3GAL5 |  |  | 180 | 0.612 | 0.2441 | Yes |
| 27 | ITM2B |  |  | 181 | 0.610 | 0.2525 | Yes |
| 28 | PLOD2 |  |  | 183 | 0.607 | 0.2604 | Yes |
| 29 | KAT2B |  |  | 193 | 0.594 | 0.2643 | Yes |
| 30 | C1orf61 |  |  | 202 | 0.587 | 0.2686 | Yes |
| 31 | SULF2 |  |  | 206 | 0.585 | 0.2753 | Yes |
| 32 | PDLIM5 |  |  | 223 | 0.572 | 0.2756 | Yes |
| 33 | PHYHIPL |  |  | 245 | 0.557 | 0.2733 | Yes |
| 34 | OMG |  |  | 252 | 0.550 | 0.2780 | Yes |
| 35 | ZNF385D |  |  | 274 | 0.531 | 0.2753 | Yes |
| 36 | LRP1B |  |  | 276 | 0.530 | 0.2822 | Yes |
| 37 | PCDH9 |  |  | 278 | 0.527 | 0.2890 | Yes |
| 38 | TENM1 |  |  | 283 | 0.524 | 0.2943 | Yes |
| 39 | ARHGAP42 |  |  | 297 | 0.511 | 0.2952 | Yes |
| 40 | BCAN |  |  | 302 | 0.510 | 0.3003 | Yes |
| 41 | ATP6V0E1 |  |  | 303 | 0.510 | 0.3074 | Yes |
| 42 | TM7SF3 |  |  | 304 | 0.510 | 0.3144 | Yes |
| 43 | SCD5 |  |  | 306 | 0.508 | 0.3209 | Yes |
| 44 | GCC2 |  |  | 309 | 0.505 | 0.3270 | Yes |
| 45 | PTPRZ1 |  |  | 310 | 0.505 | 0.3339 | Yes |
| 46 | PPP2R2B |  |  | 311 | 0.505 | 0.3409 | Yes |
| 47 | PCMTD2 |  |  | 319 | 0.501 | 0.3445 | Yes |
| 48 | IL6ST |  |  | 321 | 0.500 | 0.3509 | Yes |
| 49 | SEMA5A |  |  | 336 | 0.494 | 0.3511 | Yes |
| 50 | ITGAV |  |  | 344 | 0.490 | 0.3545 | Yes |
| 51 | ABAT |  |  | 362 | 0.483 | 0.3531 | Yes |
| 52 | CADM4 |  |  | 368 | 0.482 | 0.3574 | Yes |
| 53 | HSPA5 |  |  | 373 | 0.480 | 0.3621 | Yes |
| 54 | GATM |  |  | 375 | 0.480 | 0.3682 | Yes |
| 55 | LRRTM3 |  |  | 387 | 0.476 | 0.3696 | Yes |
| 56 | CASK |  |  | 392 | 0.473 | 0.3742 | Yes |
| 57 | VCAN |  |  | 410 | 0.466 | 0.3725 | Yes |
| 58 | SLC22A17 |  |  | 412 | 0.465 | 0.3785 | Yes |
| 59 | DPP6 |  |  | 421 | 0.462 | 0.3811 | Yes |
| 60 | EMC10 |  |  | 427 | 0.460 | 0.3850 | Yes |
| 61 | SPRY4 |  |  | 443 | 0.453 | 0.3841 | Yes |
| 62 | HSD17B12 |  |  | 453 | 0.449 | 0.3861 | Yes |
| 63 | MAP2 |  |  | 455 | 0.448 | 0.3918 | Yes |
| 64 | RFTN2 |  |  | 485 | 0.439 | 0.3840 | Yes |
| 65 | C2orf80 |  |  | 486 | 0.439 | 0.3901 | Yes |
| 66 | RAB9A |  |  | 489 | 0.438 | 0.3952 | Yes |
| 67 | SLC44A1 |  |  | 517 | 0.428 | 0.3883 | Yes |
| 68 | TNR |  |  | 548 | 0.415 | 0.3797 | Yes |
| 69 | GPM6B |  |  | 553 | 0.413 | 0.3835 | Yes |
| 70 | PMP2 |  |  | 562 | 0.411 | 0.3854 | Yes |
| 71 | GRB14 |  |  | 566 | 0.409 | 0.3896 | Yes |
| 72 | CFLAR |  |  | 569 | 0.408 | 0.3943 | Yes |
| 73 | RAB31 |  |  | 580 | 0.404 | 0.3951 | Yes |
| 74 | CPEB2 |  |  | 588 | 0.401 | 0.3973 | Yes |
| 75 | THY1 |  |  | 593 | 0.400 | 0.4009 | Yes |
| 76 | LSAMP |  |  | 611 | 0.394 | 0.3983 | Yes |
| 77 | SASH1 |  |  | 614 | 0.393 | 0.4027 | Yes |
| 78 | NTRK3 |  |  | 621 | 0.391 | 0.4053 | Yes |
| 79 | NDUFB3 |  |  | 629 | 0.389 | 0.4073 | Yes |
| 80 | TAOK3 |  |  | 631 | 0.389 | 0.4122 | Yes |
| 81 | APOD |  |  | 636 | 0.389 | 0.4157 | Yes |
| 82 | SEMA6D |  |  | 637 | 0.388 | 0.4210 | Yes |
| 83 | SEZ6L |  |  | 657 | 0.383 | 0.4173 | Yes |
| 84 | CDH10 |  |  | 669 | 0.379 | 0.4173 | Yes |
| 85 | BCHE |  |  | 677 | 0.376 | 0.4191 | Yes |
| 86 | SCARB2 |  |  | 681 | 0.375 | 0.4229 | Yes |
| 87 | FRMD6 |  |  | 703 | 0.370 | 0.4180 | No |
| 88 | RIT2 |  |  | 756 | 0.357 | 0.3982 | No |
| 89 | PLEKHH2 |  |  | 822 | 0.343 | 0.3720 | No |
| 90 | DAG1 |  |  | 834 | 0.340 | 0.3714 | No |
| 91 | PCDHGC3 |  |  | 845 | 0.337 | 0.3713 | No |
| 92 | SDC3 |  |  | 863 | 0.333 | 0.3678 | No |
| 93 | CSMD1 |  |  | 870 | 0.331 | 0.3696 | No |
| 94 | FGF12 |  |  | 873 | 0.331 | 0.3732 | No |
| 95 | ARL4A |  |  | 889 | 0.328 | 0.3706 | No |
| 96 | DST |  |  | 896 | 0.327 | 0.3722 | No |
| 97 | NCAM2 |  |  | 915 | 0.324 | 0.3681 | No |
| 98 | PEAK1 |  |  | 918 | 0.323 | 0.3716 | No |
| 99 | NKAIN4 |  |  | 923 | 0.322 | 0.3742 | No |
| 100 | USP2 |  |  | 953 | 0.317 | 0.3647 | No |
| 101 | SLITRK2 |  |  | 967 | 0.315 | 0.3629 | No |
| 102 | ITGB8 |  |  | 970 | 0.314 | 0.3663 | No |
| 103 | NOMO1 |  |  | 973 | 0.314 | 0.3697 | No |
| 104 | ALDH6A1 |  |  | 987 | 0.311 | 0.3678 | No |
| 105 | EVI5 |  |  | 994 | 0.310 | 0.3692 | No |
| 106 | NTN1 |  |  | 1004 | 0.309 | 0.3692 | No |
| 107 | SIRT2 |  |  | 1018 | 0.307 | 0.3673 | No |
| 108 | SERPINE2 |  |  | 1030 | 0.304 | 0.3662 | No |
| 109 | SCRG1 |  |  | 1032 | 0.304 | 0.3699 | No |
| 110 | PELI1 |  |  | 1059 | 0.298 | 0.3617 | No |
| 111 | RAP2A |  |  | 1067 | 0.297 | 0.3624 | No |
| 112 | CD9 |  |  | 1070 | 0.297 | 0.3656 | No |
| 113 | LINGO1 |  |  | 1074 | 0.297 | 0.3683 | No |
| 114 | LYPD1 |  |  | 1094 | 0.293 | 0.3633 | No |
| 115 | DNER |  |  | 1104 | 0.292 | 0.3630 | No |
| 116 | LAMA4 |  |  | 1114 | 0.290 | 0.3627 | No |
| 117 | PIK3R1 |  |  | 1121 | 0.289 | 0.3639 | No |
| 118 | KCND2 |  |  | 1151 | 0.285 | 0.3540 | No |
| 119 | CACNB2 |  |  | 1155 | 0.285 | 0.3565 | No |
| 120 | STXBP3 |  |  | 1164 | 0.283 | 0.3566 | No |
| 121 | SEPT8 |  |  | 1167 | 0.283 | 0.3596 | No |
| 122 | KLHL5 |  |  | 1226 | 0.276 | 0.3358 | No |
| 123 | UNC80 |  |  | 1227 | 0.276 | 0.3396 | No |
| 124 | PTPRE |  |  | 1243 | 0.275 | 0.3362 | No |
| 125 | STARD4 |  |  | 1254 | 0.274 | 0.3352 | No |
| 126 | SGK1 |  |  | 1283 | 0.270 | 0.3257 | No |
| 127 | TMEM206 |  |  | 1285 | 0.270 | 0.3289 | No |
| 128 | NLGN3 |  |  | 1292 | 0.269 | 0.3298 | No |
| 129 | SSR1 |  |  | 1300 | 0.268 | 0.3301 | No |
| 130 | CTTNBP2 |  |  | 1350 | 0.262 | 0.3104 | No |
| 131 | SNX7 |  |  | 1363 | 0.261 | 0.3083 | No |
| 132 | KCND3 |  |  | 1365 | 0.261 | 0.3114 | No |
| 133 | LINC01105 |  |  | 1377 | 0.259 | 0.3098 | No |
| 134 | WSCD1 |  |  | 1401 | 0.256 | 0.3024 | No |
| 135 | PDE4B |  |  | 1404 | 0.256 | 0.3050 | No |
| 136 | GLDC |  |  | 1425 | 0.255 | 0.2989 | No |
| 137 | KANK1 |  |  | 1449 | 0.252 | 0.2915 | No |
| 138 | BLM |  |  | 1507 | -0.262 | 0.2680 | No |
| 139 | NDUFAF2 |  |  | 1557 | -0.274 | 0.2484 | No |
| 140 | PDGFRA |  |  | 1601 | -0.286 | 0.2319 | No |
| 141 | ILDR2 |  |  | 1650 | -0.300 | 0.2132 | No |
| 142 | TRIO |  |  | 1661 | -0.304 | 0.2126 | No |
| 143 | NOC3L |  |  | 1717 | -0.324 | 0.1909 | No |
| 144 | PHLDA1 |  |  | 1718 | -0.324 | 0.1954 | No |
| 145 | DSEL |  |  | 1735 | -0.333 | 0.1924 | No |
| 146 | NUP50 |  |  | 1823 | -0.376 | 0.1562 | No |
| 147 | CHD7 |  |  | 1874 | -0.400 | 0.1379 | No |
| 148 | LIMA1 |  |  | 1899 | -0.421 | 0.1323 | No |
| 149 | E2F3 |  |  | 1907 | -0.429 | 0.1349 | No |
| 150 | DENR |  |  | 1956 | -0.457 | 0.1183 | No |
| 151 | HAS2 |  |  | 1968 | -0.464 | 0.1195 | No |
| 152 | FABP5 |  |  | 2033 | -0.523 | 0.0963 | No |
| 153 | EPB41L2 |  |  | 2067 | -0.576 | 0.0885 | No |
Table: GSEA details [plain text format]

  

Fig 2: RICHARDS\_NOWAKOWSKI\_SCIENCE\_2017\_OPC\_UPREG: Random ES distribution      
 Gene set null distribution of ES for **RICHARDS\_NOWAKOWSKI\_SCIENCE\_2017\_OPC\_UPREG**
